# Feature Space Transformation for Fault Diagnosis of Rotating Machinery under Different Working Conditions

**DOI:** 10.3390/s21041417

**Published:** 2021-02-18

**Authors:** Gye-Bong Jang, Sung-Bae Cho

**Affiliations:** Department of Computer Science, Yonsei University, Seoul 03722, Korea; gyebong.jang@yonsei.ac.kr

**Keywords:** fault diagnosis, domain adaptation, attention mechanism, feature space transformation, gearbox, vibration measurement

## Abstract

In recent years, various deep learning models have been developed for the fault diagnosis of rotating machines. However, in practical applications related to fault diagnosis, it is difficult to immediately implement a trained model because the distribution of source data and target domain data have different distributions. Additionally, collecting failure data for various operating conditions is time consuming and expensive. In this paper, we introduce a new transformation method for the latent space between domains using the source domain and normal data of the target domain that can be easily collected. Inspired by semantic transformations in an embedded space in the field of word embedding, discrepancies between the distribution of the source and target domains are minimized by transforming the latent representation space in which fault attributes are preserved. To match the feature area and distribution, spatial attention is applied to learn the latent feature spaces, and the 1D CNN LSTM architecture is implemented to maximize the intra-class classification. The proposed model was validated for two types of rotating machines such as a dataset of rolling bearings as CWRU and a gearbox dataset of heavy machinery. Experimental results show the proposed method has higher cross-domain diagnostic accuracy than others, therefore showing reliable generalization performance in rotating machines operating under various conditions.

## 1. Introduction

Heavy equipment is commonly employed at large construction sites such as mines and quarries. Its failure directly affects productivity, which can cause great losses to both customers and corporations. Therefore, identifying eminent failures in advance and minimizing downtime are essential for both manufacturers and customers. The advent of the Industry 4.0 era has also increased demand for diagnosis and prognostics with use of smart sensors. Under the slogan “Industry 4.0”, the development of intelligence applications is accelerating at various industrial sites [1]. Fault diagnostics for parts susceptible to damage such as sun, planetary, and ring gears—major components of heavy machinery—rely on detecting and monitoring changes in the magnitude of fault frequency. However, the complex kinematics of planetary gearboxes generate complex vibration signals, making it difficult to identify characteristic error frequencies. A failure in a planetary gearbox can shut down the entire vehicle, resulting in major economic losses and even human casualties. Condition monitoring and initial fault diagnosis aim to prevent accidents and save costs for planetary gearbox users. 

Machine learning technology has had a number of successes in the field of fault diagnosis in recent years. Previous studies tended to consider autoencoders (AEs), convolutional neural networks (CNNs), and recurrent neural networks (RNNs) [2,3]. RNNs are a type of deep learning architecture designed for time-series data—i.e., data in which the current output is heavily dependent on the data that preceded it, such as in language. Generally, output from the current time step comprises all or part of the input for the next time step operation [4]. However, early RNNs had a problem of quickly forgetting the impact of previous data after only a few iterations (vanishing gradient problem). Long Short-Term Memory (LSTM) networks were developed to solve this problem [5]. Hence, most modern RNNs are LSTM implementations and are extensively used to identify gradual and time-dependent machine faults [6,7]. In particular, fault diagnosis for a rotating component, an essential aspect of various applications, has received a lot of attention in industry. For bearing failure diagnosis, a method of indirectly using the starter current rather than data collected from a rotating body was proposed [8]. In addition, the feature extraction method based on pre-learning, which can more robustly extract the fault features of a rotating body, has been successfully applied [9].

However, most existing studies are generally conducted under the assumption that the source and target data have similar distributions [10]. This means that training and testing data must be obtained using the same equipment under the same working conditions. Unfortunately, in general, the operating speed or load of an actual rotating body will constantly change, creating an abnormal vibration signal and error frequency with time-varying characteristics, making fault diagnosis more complicated. Therefore, this assumption is difficult to meet in practice and variations in operating conditions create significant disparities in the distribution of the target domain data [11,12]. As a result, the learned fault diagnosis knowledge does not generalize well in the test area due to domain shift issues. A method of solving these problems that has recently attracted attention is transfer learning, which transfers knowledge between different areas [13,14,15]. Transfer learning methods can be summarized into four main categories: (1) Instance-based transfer learning, which rearranges the weights of the learning model through retraining with the target data; (2) feature-based transfer learning to find domain-invariant features by reducing the distribution mismatch between the source and target domains; (3) relationship-based transfer learning, which transfers mutual knowledge based on the similarity between the interactions of two domains; and (4) in model-based transfer learning, in which parameters are transferred directly or fine-tuned by a classifier for the target domain. 

In this paper, we propose a new domain transformation-based diagnosis method for diagnosing cross-domain failures of rotating machinery. Label data collected under specific operating conditions and normal state data collected under different operating conditions are used for model training. To emphasize the spatiotemporal information of the input signal, the input signal is preprocessed using short-term Fourier transform (STFT). To minimize the distribution mismatch between the source domain and the target domain, we propose a semantic transformation algorithm in the latent space. 

For feature extraction, a deep convolutional neural network with an attention mechanism is adopted, and a domain shift algorithm is introduced to match the distribution of data across the domain. The results indicate that the proposed method is an effective and promising tool for diagnosing cross-domain defects in gearboxes. The main contributions of this paper are as follows:

- A domain transformation method is proposed for cross-domain fault diagnosis in rotating machines with significantly changing operating conditions.
- A feature extraction method is proposed that can focus on features related to failures using STFT and attention mechanisms.
- The results of the preferred dataset and the actual dataset prove the generalization performance of the proposed method.

In the experiment, two types of dataset of a gearbox system, such as benchmark and real machine data, are used for validation. Both datasets contain various types of failures collected under different working conditions. 

The paper is organized as follows: Section 2 describes related works. Section 3 defines the sensing data and provides an overview of the learning model. Section 4 proposes an in-depth learning diagnosis method to extract fault relevant features, also with a domain space shifting method. Section 5 demonstrates the validity of the proposed approach and performance in two cases: a benchmark of a popularly used dataset and real equipment dataset. Section 6 concludes the paper.

## 2. Related Works

Among the various transfer learning methods, the domain adaptation technique has been widely adopted for fault diagnosis by assuming the same labeling operation for the training and test data [16]. In general, domain adaptation approaches aim to extract domain invariant features even if domains are moving. In particular, a deep learning-based domain adaptation method that utilizes both powerful and transfer learning capabilities has been successfully developed [17,18,19]. 

To diagnose defects in rolling bearings, a domain adaptation method was proposed that improved the generalized class distance [20]. In addition, a domain adversarial learning system is proposed, since adversarial training is a model for learning generalized features across domains (DCTLN) [21].

Some researchers minimize domain differences between different working conditions through maximum mean mismatch (MMD). MMD is a distance-based standard method for minimizing discrepancies between two datasets [22,23]. In addition, a multi-kernel based MMD has been proposed [24,25]. Unlike traditional distance-based methods (e.g., Kullback–Leibler divergence), an MMD can estimate nonparametric distances and does not require calculating the median density of the distribution [26]. It has been proposed to use an auto-encoder to project into the function shared subspace, and MMD distances are used to minimize the inter-domain distance. Sufficient target data, not labeled via the automatic encoder, also contribute to the learning of the function [27].

However, if the number of parameters of data required for training is small or difficult to obtain, training itself cannot proceed. Therefore, minimizing the MMD distance cannot be guaranteed to secure a common set of authentication functions for fault diagnosis. To overcome these deficiencies, domain adversarial neural networks (DANNs) have been proposed [28]. 

A DANN introduces a gradient inversion layer to extract features that can determine where the target domain is similar to the source domain. The Adversarial Identification Area Adaptation (ADDA) method extends the Adversarial Domain Adaptation method to provide a generalized view [29]. The Conditional Domain Adversarial Network (CDAN) method was inspired by the Conditional Generative Adversarial Network (CGAN) and used several linear conditions to capture the cross covariance between class prediction and feature expression [30]. Adversarial learning, which extracts domain invariant feature expressions through the adversarial training of feature extractors and domain classifiers, can achieve better adaptability than most MMD-based methods [31,32].

However, the issue of modal stability in adversarial training remains [33,34]. In theory, the adversarial training mode is expected to reach equilibrium to extract domain-invariant feature representations. However, if the data distribution varies significantly across domains, it is difficult to scale down the domain adaptation model. When diagnosing failures in rotating machines, the data distribution is greatly affected by the working conditions.

As described above, both the distance-based approach and the adversarial learning approach have a common problem that they require sufficient data from the target domain. Therefore, we propose a domain movement method that uses normal data of the target domain, which are relatively easy to collect, to reflect realistic conditions.

## 3. Preliminaries

**Figure 1:**
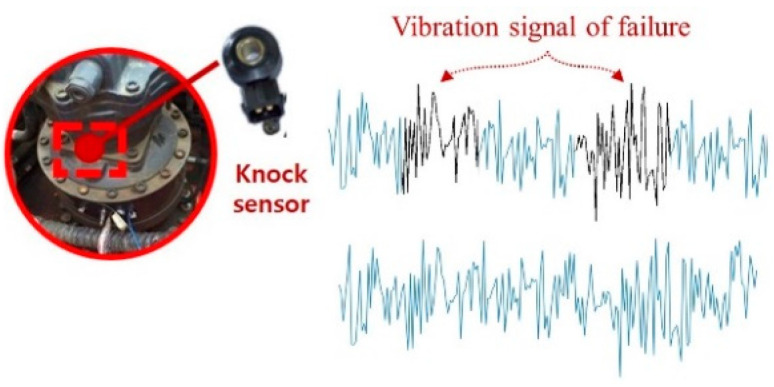
Sensor mounting location and measurement vibration signals.

Gearbox vibrations have a very complex structure. Figure 1 shows the location of a typical sensor to measure vibrations in heavy equipment. The sensor is mounted outside the gearbox and collects not only gearbox vibration but also vibration (noise) from other equipment assets. We intentionally used a low-cost knock sensor commonly deployed in automotive engines. These have only a moderate measurement range and resolution, which may incur noise or missing values at the sensor. These sensors are typically used in real equipment, as they are much cheaper and less capable than those used in academic experimental environments. The specifications of the vibration sensor are described in Table 1. Vibration data were collected by the control unit with an analog-to-digital converter.

### 3.1. Measurement Mechanism for the Vibration Signal

For the dataset of the real equipment used in the experiment, the vibration signal was measured using a knock sensor at a 25 kHz sampling rate. Since there was no tachometer to measure the rotational speed, vibrations were measured at high frequencies. The data measured from the actual equipment were collected under the same conditions running in the field. To verify the failure type, the same type of failure was reproduced as that used for training. 

**Figure 2:**
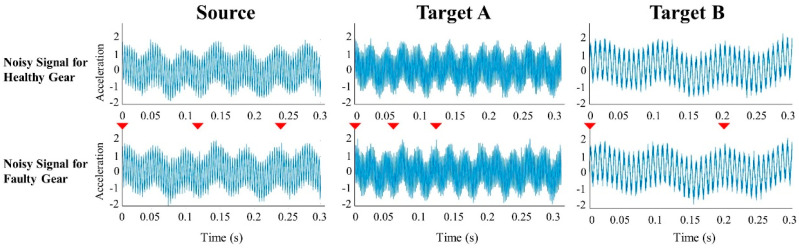
The vibration signal for each status in various operation conditions.

Defect areas are difficult to determine, because defect signals are measured irregularly at various rotation speeds. Figure 2 shows the measured vibration signals for each status. Depending on the operating condition, the pattern of the fault signal can change greatly; in the case of a specific fault, it has a subtle change like an impulse signal. 

### 3.2. Overall Idea

**Figure 3:**
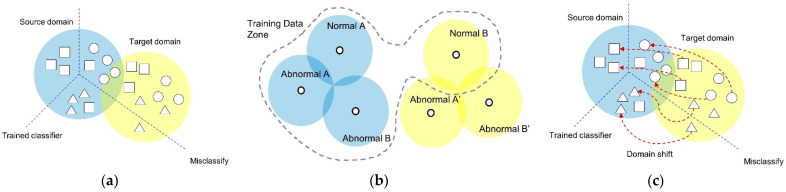
Visualization of the distribution concept for each domain dataset. (**a**) Before domain transformation; (**b**) the goal of domain adaptation; (**c**) training dataset of the proposed model.

We have a labeled dataset $\left\{X_{S},Y_{S}\right\}$ extracted from the source domain, $\left\{X_{T}^{N}\right\}$ from the target domain and unlabeled target domain data $\left\{X_{T}\right\}$. Figure 3 conceptually describes the domain shifting problem. Domain shifting due to noise interference and fluctuations in the working conditions can significantly degrade the classification performance in the target domain.

Generally, applying a trained model to a new environment requires a new way of generalizing information from the new domain. Inconsistency with new data can be minimized through distance-based learning between available data by active research in recent machine learning-based research [35]. This distance-based discrepancy learning method has been applied successfully in many research tasks such as human activity recognition and human re-identification [36,37].

All the above-mentioned studies assume that the target domain can collect enough data for learning. However, it may not be realistic in a real-world setting, because collecting fault data takes a lot of time and money. On the other hand, normal data can be more easily collected from other domains. Therefore, we propose a method of solving the domain mismatch through a learning method that only includes normal data of the target domain. Inspired by Natural Language Processing called "word embedding", we propose a method of moving feature spaces between domains.

We applied a reconstruction-based stacked autoencoder model that can represent the input signal in a low-dimensional shared space to move the feature vectors of the input data in space. An autoencoder is a deep learning architecture that can efficiently code data. The latent space learned by an autoencoder is that which can best compress and express the features of data; it was proposed to solve the domain adaptation problem by adjusting the latent space.

### 3.3. Short-Time Fourier Transform (STFT)

The frequency characteristics of a signal can be investigated based on a Fourier series, Fourier Transform, and Discrete Fourier Transform (DFT). STFT is used in the frequency tracking of a tacholess system to represent the spectrogram of the signal. The vibration signals of mechanical systems are often non-stationary. Hence, there is a need for tools for the analysis of time-based frequency content. FFT and DFT allow the investigation of a signal immediately, and temporal-specific information is lost. STFT allows the computation of multiple frequency spectrums by performing successive DFT on a windowed signal. Therefore, it adds a new dimension, defined by

(1) $$S\left(f,\tau \right)=\int_{-\infty }^{\infty }x\left(t\right)w\left(t-\tau \right)e^{-j2\pi ft\;}dt$$

where w(t−τ) is the window that moves along the signal x(t). In practice, STFT is used to compute the spectrogram of the signal. It is a time-frequency map where the square of the amplitude ${\left|S\left(f,\tau \right)\right|}^{2}$ is plotted over frequency *f* and time *τ*. Even though it is a simple tool, there is a trade-off between the time and frequency resolution, which relate to each other as

(2) $$△f=\frac{1}{△t}$$

where △f and △t are the frequency and the time resolutions, respectively. Therefore, striving for a better time resolution could reduce the frequency accuracy and vice versa. In this paper, the window size was defined as 512 and the overlapping size as 128.

### 3.4. Attention Mechanism

An attention mechanism is proposed to enable learning the alignment between the source and the target tokens to improve the neural machine translation performance [38]. Attention mechanisms have mainly been used in language and image fields to find focused words and images, and several studies have used basic and modified attention mechanisms for time series. LSTM using an attention mechanism is proposed for multivariate time series, employing the following attention mechanism transition functions.

(3) $$M=tanh\left[\begin{array}{c} W_{h}H\\ W_{v}v_{a}\cdot e_{n} \end{array}\right]$$

(4) $$\alpha =softmax\left(w^{T}M\right)$$

and

(5) $$r=H\alpha^{T}$$

where H = $\left\{h_{1},h_{2},\ldots ,h_{n}\right\}$ is a matrix containing features $h_{i}$ extracted by the prediction model, $e_{n}\in R^{n}$ is a vector of ones, α is a vector of attention weights for features in $H;\;v_{a}$ is the embedding aspect for the attention mechanism, and r is the output from the attentive neural network as weighted features H [39].

## 4. Proposed Model

**Figure 4:**
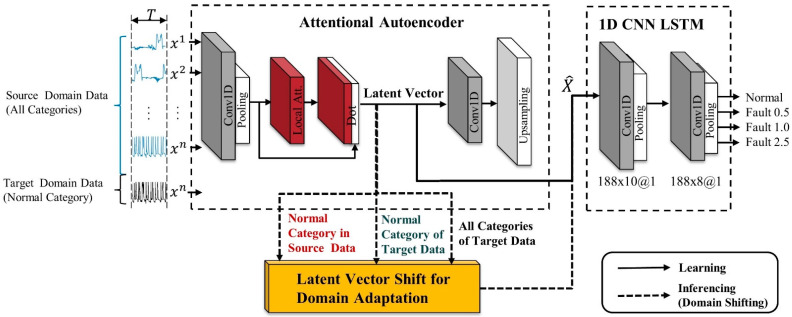
The architecture of the proposed model including a latent vector shifting algorithm.

We extracted time and space information from the original signal through STFT pre-processing and classified the fault type from the input signal using an autoencoder with attention mechanism and 1D CNN LSTM classifier. In addition, we used feature space transformation for domain adaptation in this paper to improve the cross-domain classification performance. Bearing vibration signals collected from a knock sensor are usually 1D, so it is recommended to use 1DCNN for vibration signal processing. In addition, most of the physical failure signals are generated by impulse signals with periods generated by specific gears or bearings [40]. Therefore, a learning model is designed to localize the impulse signal with 1DCNN and extract contextual features with LSTM. Figure 4 shows the architecture of the learning and domain transformation algorithm. The whole model consists of an attentional autoencoder for latent vector representation and a 1D CNN LSTM-based classifier for classifying failure types from latent vectors. In the inference stage, a latent vector shift stage is added to transform the input data.

### 4.1. Understanding Latent Space

**Figure 5:**
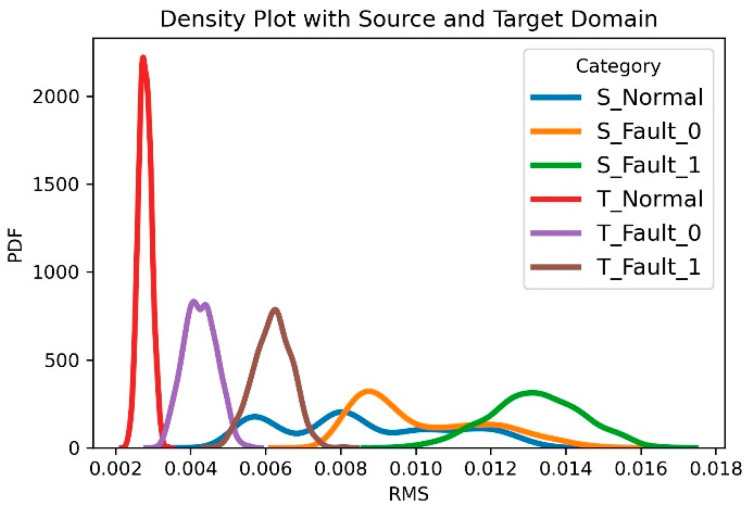
A data distribution plot for the failure modes of a gearbox with two working conditions in a real machine dataset. The source domain data is denoted S_Normal, S_Fault_0, S_Fault_1; the target domain data is denoted T_Normal, T_Fault_0, and T_Fault_1.

Figure 5 shows the data distribution of three statuses under two different working conditions. We can confirm that the distribution between the source domain and the target domain is significantly different. However, we can see that the alignments of the distributions for each category are similar. Based on the above logic, the model trained on the source domain data encodes the target domain data around the most similar features. Therefore, the target domain alignment does not deviate from the source domain alignment. 

Therefore, the weights of a deep neural network are divided into two categories such as basic projection and specific projection. The weight of the base projection is to extract the base features from the input data. Although the basic features are different, they are the basic components involved in the distribution of data and similar. Conversely, the weights of a particular projection can learn many representative features for each category. Therefore, features are extracted to follow a similar shape for newly collected data due to the default projection weights. We try to distribute the feature spatial distribution of newly collected data as similarly as possible to the previously learned data. Therefore, by calculating a specific projection weight that includes the difference between the domains from the normal data, we can reduce the difference between the two by moving the domains [41].

### 4.2. Encoder with Spatial Attention

**Figure 6:**
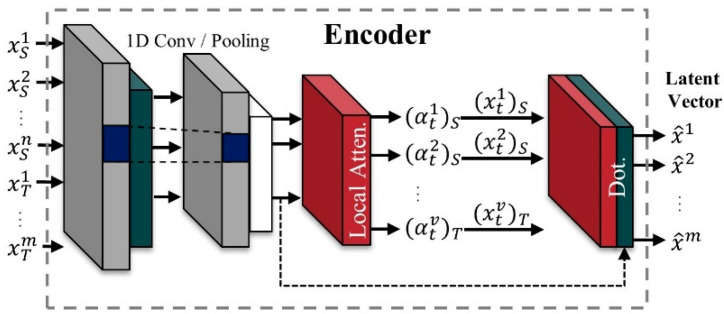
Attention mechanism-based encoding module.

We propose using an autoencoder with an attention mechanism model to represent the features that focus on failure-relevant signals in the latent space. A spatial attention layer for the proposed autoencoder model is inserted between each layer in the encoder, as shown in Figure 6. Each attention score is applied in the next layer using its dot product with the input vector. The autoencoder is an unsupervised model that learns data representation by generating outputs that are similar to its inputs. Inputs are encoded in latent representation by a high-density layer.

Finally, the latent representation is passed to the decoder to restore back to the same characteristic dimension as the original input. Autoencoder models are generally well-known for their use in noise cancelling and data interpolation [42,43]. Therefore, by leveraging the two features of an autoencoder, we devised a more powerful feature representation method that combines the attention mechanisms. 

Inspired by [44], the average pooling and max pooling are performed through the channel dimension and then a 1 × 1 convolution is applied to feature representation. In $\alpha_{t}^{m}=\sigma_{v}\left(Mean\left(x_{t}^{m}\cdot {\left(x_{t}^{m}\right)}^{T}\right)\right)$, $\sigma_{v}$ is a softmax activation function. Here, the role of the local attention is to localize the fault signals from the raw signal and ${\hat{x}}_{t}^{m}=\left[\alpha_{t}^{m};x_{t}^{m}\right]\in {\mathbb{R}}^{m+m}$. In addition, as described above, a context vector is the output obtained from the dot-product attention score function using the input signal. Using the proposed input attention mechanism, the encoder can selectively focus on certain input series instead of treating all input series equally. 

**Figure 7:**
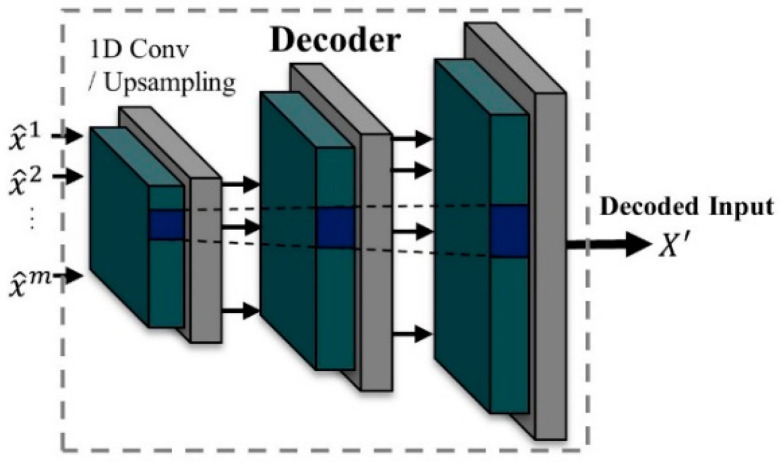
Decoding process for reconstructing the input.

The decoder reconstructs the latent vector into a similar signal to the input, as shown in Figure 7. The decoding process operates in reverse order of the encoding. Furthermore, the attention layer is not included in the decoder for the purpose of stable learning.

The reconstruction error is

(6) $$L=\left|\alpha^{m}\cdot x^{m}-{\left(x’\right)}^{m}\right|$$

The output of the dot product of the attention score and the input layer emphasizes the part on which the model should focus. In addition, Algorithm 1 describes the training procedure of the autoencoder.

**Algorithm 1** Autoencoder Training
**Input:** Input data $X=x_{1},x_{2},\cdots ,x_{n},$ number of epochs n, learning rate λ, amount of batches T.
θ={ω,b} is the parameters of an autoencoder.
**Output:** Trained model *E, D*, latent vector E(·).
1: **begin**2:  Initialize parameters for *E, D*3:  **for** t=1→n **do**4:  # extract latent vector and reconstruction at each timestep5:   **for** i=0→T **do**6:    $\alpha_{i}=\sigma \left(Mean\left(x_{i}\cdot x_{i}^{T}\right)\right)$7:    ${\tilde{x}}_{i}=\alpha_{i}\cdot x_{i}$8:    ${\hat{x}}_{i}=\;\sigma \left(\omega \cdot {\tilde{x}}_{i}+b\right)$9:    Compute the reconstruction loss according to (6)10:    $ℒ\left({\tilde{x}}_{i},{\hat{x}}_{i}\right)=\overset{T}{\underset{i=1}{\sum }}\left[x_{i}log{\hat{x}}_{i}+\left(1-x_{i}\right)\log\left(1-{\hat{x}}_{i}\right)\right]$11:    Compute the gradient of the loss with respect to θ12:    **for** $\theta_{i},g_{i}$ **do**13:    $\theta_{i}=\theta_{i}-\lambda \cdot g_{i}$14:    **end for**15:   **end for**16:  **end for**17: **end procedure**


### 4.3. Classifier Based on 1D CNN LSTM

**Figure 8:**
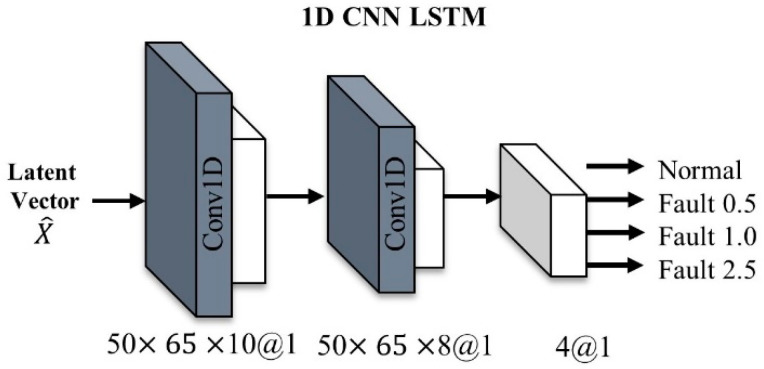
The classifier based on 1D CNN LSTM.

Figure 8 shows the conceptual architecture of a classifier. In the classifier, the 1D CNN LSTM structure was adopted to learn the contextual features. The architecture consists of 10 layers, including two convolutional layers, two 1D CNN LSTM layers with kernel sizes at 10 and 8, respectively, two batch normalizations with dropout layers, and three dense layers. 

The size of the latent vector taken for the data preprocessing process is (None, 50, 65, and 8), and then the output shape changes into (None, 50, 65, and 10). The second layer is the CNN LSTM layer. There are 64 neurons in this layer, and the output shape of this layer is (None, 50, 65, 64). The next layers consist of batch-normalization and dropout, and the above layers are repeated twice. The main purpose of having a dropout layer is to reduce over-fitting, and a softmax layer is used at the end for classification.

### 4.4. Domain Transformation on Latent Space for Inference

We describe how to use Neural Linear Transformation (NLT) model to allow “domain transformation” in this section. The goal of domain transformation is to transfer the target domain to the source domain distribution to successfully utilize the trained model. In the word embedding area, vector representation is usually used for word analogies to embed a word (i.e., Kings and queens magic).

(7) “King−Man+Woman≈Queen”

**Figure 9:**
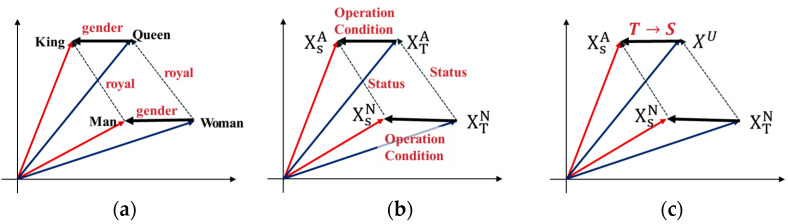
2D representation of the semantic analogies. Word analogies for (**a**) vector operation, (**b**) latent vector operation, and (**c**) latent vector operation of unknown input data.

According to this vector operation, we can assume that a vector representation of the word “queen” is possible if the words “king”, “woman”, and “man” are known. Figure 9 shows the 2D representation of the semantic analogies. 

Inspired by word inference, we propose an algorithm to allow for transformation between domains.

(8) $$X_{S}^{N}-X_{T}^{N}\approx X_{S}^{A}-X_{T}^{A}$$

where $X_{S}^{N},\;X_{S}^{A}$ is the encoded vector of the normal and abnormal states in the source domain, respectively, and $X_{T}^{N},\;X_{T}^{A}$ is the encoded vector of the normal and abnormal states in the target domain, respectively. These data are encoded through the proposed autoencoder. Thus, these data are represented in latent space. Abnormal means a dataset of whole failure status $X_{T}^{A}=\left\{X_{T}^{F_{1}},X_{T}^{F_{2}},\ldots ,X_{T}^{F_{n}}\right\}$, where n is the number of failure cases. To apply the concept of transformation by inserting a vector into a word inference as seen above, a feature expression method capable of expressing the source and target data in a specific space is required. Therefore, our proposal aims to move the data of the target domain by constructing a latent space containing the normal state data of the target domain through an attentional autoencoder structure.

In Figure 9, the vector operation for moving according to the operating condition (domain) of the machine is as in Equation (8). However, since we do not know $X_{T}^{A}$, the domain shifting algorithm is required to transfer $X_{T}^{U}$ to $X_{S}^{N}$, which is derived as shown in Equation (9).

The target domain data is denoted as $X^{U},$ because we do not know the state of the target domain. Moving the target domain data to the source domain area of the learned latent space is done through the Equation (9) operation. However, $X^{U}$ is not from the source domain dataset $X^{U}\notin X_{S}$.

(9) $$T\to S:\;X^{U}+X_{S}^{N}-X_{T}^{N}$$

where $X^{U}$ means the input dataset. According to (9), depending on whether $X^{U}$ is normal or abnormal, the space is moved as follows.

(10) $$\{\begin{array}{cc} X^{U}=X_{T}^{N}\;,\; & X^{U}\to X_{S}^{N}\\ X^{U}=X_{T}^{A}, & X^{U}\to X_{S}^{A} \end{array}$$

The domain shift function $f_{d_{1}\to d_{2}}$ is defined as the following:

(11) $$f_{d_{1}\to d_{2}}\left(x_{t},\mu \left(x_{s}\right),\mu \left(x_{t}\right)\right)=x_{t}+\mu \left(x_{s}\right)-\mu \left(x_{t}\right)=x_{s}$$

where $x_{t}$ and $x_{s}$ are the input and the transferred data that follow the distributions of the target and the source domain, respectively. μ denotes the average value of the unary function. The $\mu \left(x_{s}\right)-\mu \left(x_{t}\right)\;$ indicates a vector representing the direction from the target domain to the source domain. Therefore, $f_{d_{1}\to d_{2}}$ shifts the input data $x_{t}$ from the target domain to the source domain, yielding the shifted latent vector that follows the distribution of the source domain.

## 5. Experiments

To verify the performance of the proposed technique, experiments were conducted with two fault diagnosis cases. The first case is an open benchmark data case for rolling element bearing diagnostics. The second was for the fault diagnosis of a rotating gearbox of heavy equipment.

We compared the proposed model with the state-of-the-art classification and CNN architectures, which included the Deep Convolutional Transfer Learning Network (DCTLN) [21], Deep Convolutional Neural Networks with Wide First-layer Kernels (WDCNN) [45], Domain-Adversarial Training of Neural Networks (DANN) [28], and Discriminative Adversarial Domain Adaptation (DADA) [46]. For DANN [28] and DADA [43], the data format is 2D, so Case 2 data was modified with 2D convolution. The detailed parameters for each experiment are shown in Table 2. To verify the feasibility and practicality of the heavy equipment failure diagnosis, the experiment for Case 2 was set to the same as the actual operating conditions, including noise and other environmental factors. Table 3 shows the parameters of the encoder. We repeated each experiment five times and have reported the average and the standard deviation of the accuracy.

### 5.1. Case Study 1: CWRU Dataset

The Case Western Reserve University (CWRU) bearing dataset is a benchmark dataset collected under various operating conditions and was used to verify the performance of the proposed method.

**Figure 10:**
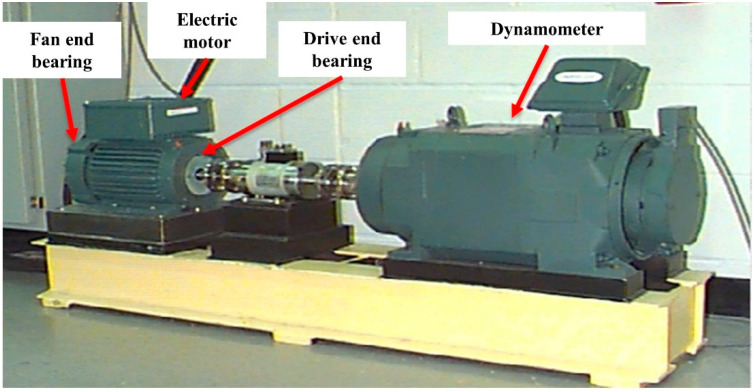
Measuring position of each vibration signal in the CWRU dataset.

In particular, the defect data of the 12 k drive end bearing was selected as experimental data [47]. There are four types of bearing fault locations: normal, ball fault, inner race fault, and outer race fault. Each error type was available in three sizes, 0.007, 0.014, and 0.021 inches, respectively, so there are a total of 10 types of error labels. Each fault label contains three types of driving conditions, 1772, 1750, and 1730 RPM motor speed (1, 2, and 3 hp), respectively. Each sample was extracted from a single vibration signal as shown in Figure 10.

We defined 70% of the vibration signal as a training sample and the rest as a test sample. As shown in Table 4, each dataset was under different operating conditions, with loads of 1, 2, and 3 hp, respectively. Each dataset contained 12,540 training samples and 3140 test samples, respectively. As shown in Table 5, the CWRU dataset has a total of 10 classes, and each fault is divided according to fault’s location and size. One of each dataset is defined as the source data, and one of the other datasets is defined as the target domain.

The proposed model performed similarly to or better than the state-of-art method in most cases, as shown in Table 6. From the results, we found the following interesting fact: when learning from the data obtained from a device rotating at high speeds, the evaluation of low-speed data was quite satisfactory. However, when evaluating the data obtained from a device rotating at low to high speed, reliability was slightly lower.

### 5.2. Case Study 2: Real Machine Dataset

Unlike the CWRU dataset, the equipment data were collected from heavy equipment actually operating under various conditions (speed, load, etc.) and exposed to different noise environments, taking into consideration its complex and large structures. By doing so, we demonstrated that our model remains robust even in a wide range of speeds and in environments with high noise levels. The corresponding data does not contain accurate speed information (measured without using a tachometer), and the tests were carried out at the speeds of about 100%, 75%, 50%, and 25% based on user manipulation. In addition, data were collected for some classes but not for all classes. Table 7 shows the operating conditions and fault classes of the collected data. One of each dataset is defined as the source data, and one of the other datasets is defined as the target domain.

**Figure 11:**
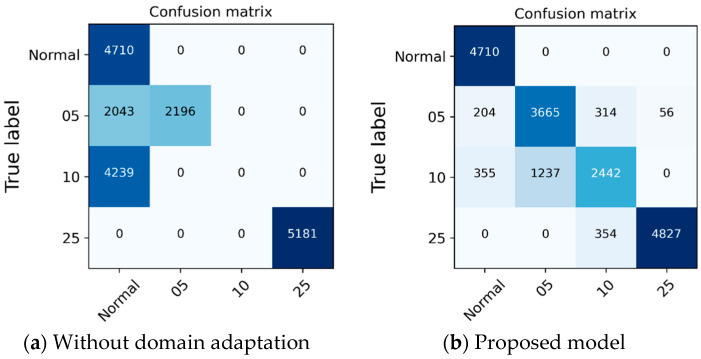
The confusion matrix of the classification results of the proposed model or without attention model.

The domain data from the new working condition, measured from the actual machine, was used throughout the entire experiment. The proposed model performed better than the state-of-art method in most cases, as shown in Table 8. The confusion matrix for the experimental results is shown in Figure 11. Approximately 79~83% accuracy was achieved for the target domain data. Moreover, we were able to confirm that the ability to recognize the minimum failure (Abnormal_0.5/0.5 mm) and the abnormalities were well distinguished.

**Figure 12:**
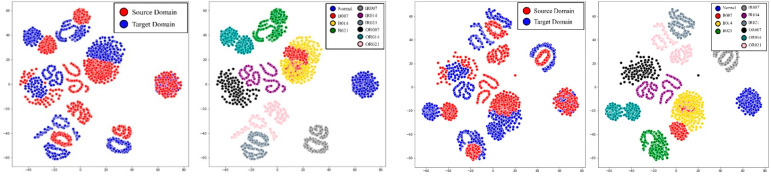
The t-SNE visualization of the feature at the latest hidden layer for each domain data, such as the source and target, and each class in CWRU.

**Figure 13:**
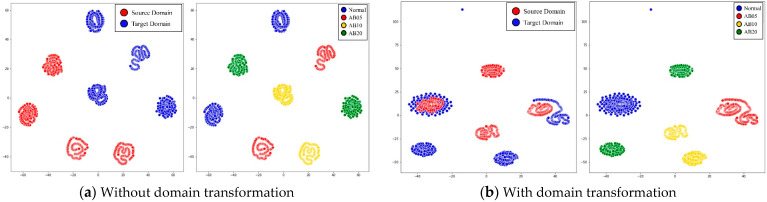
The t-SNE visualization of feature at latest hidden layer for each domain data such as source and target and each class in real machine.

In all cases, the proposed model outperformed other models and proved to be an effective technique for diagnosing failures in a rotating body. Figure 12 is the results of comparing the effects of the proposed model through the t-SNE results of the A→B transformation case of the CWRU dataset. Before the domain transformation is applied, the source domain and the target domain are clearly separated, but through domain transformation, the failure data of the target domain not used for learning also shares the source domain and the distribution area. In Figure 13, it can be seen that the domain distribution is similarly close to the actual equipment data in the A→B transformation case. However, in the case of the green area in Figure 13b, it appears that the distance is not close. Considering that the fault classification performance shows high results, it is judged that it is located in the area where the fault can be identified. In addition, since CWRU data is data acquired through limited experiments in a laboratory environment, there is no significant difference in discrepancy between the source and target domain data. However, in the real equipment case, we found that the distances between the distributions are unpredictable and markedly different. Nevertheless, it is encouraging that the proposed model can achieve the desired performance.

### 5.3. Evaluation on A Real Machine

**Figure 14:**
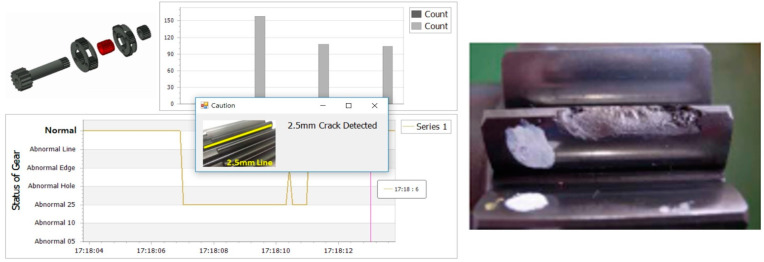
Verification on a real machine using the embedded software that included the proposed learned model.

We used data collected from the actual equipment to verify reliability of the proposed learning model. Since we did not know the real operating conditions, the raw data looked like unseen data that contained various trends. Figure 14 shows a program developed to evaluate the data received at 1500 Hz in real time and to diagnose the most frequently occurring actual fault conditions. The system used occurrence count as a criterion to minimize false alarms.

We physically disassembled the gearbox of the machine, compared the estimation type of the program with the actual failure type, and obtained the same results as the failure type estimated by the program. Therefore, the proposed system is suitable for practical applications and has great significance in the large-scale mining machinery industry.

## 6. Conclusions

This paper presents a domain transformation method for diagnosing faults in rotating machines operating under various operating conditions. The proposed algorithm successfully performs domain transformation using only the general data of the target domain to solve real problems for which it is difficult to obtain failure data under various conditions. An autoencoder with an attention mechanism was applied to extract features containing relevant fault information, and a new latent vector transformation method inspired by word embedding was proposed.

The proposed model was verified with widely used public data and data collected from real equipment and showed an accuracy above 83%, resulting in a significant performance improvement over the existing method. In addition, the proposed model was mounted on an embedded board and verified under actual equipment operation, demonstrating that it can effectively diagnose faults in a new environment. Attempts to apply the system to monitor actual excavator operation have been successful, and the system accurately identified existing defect cases without prior knowledge. Therefore, the proposed system is suitable for practical applications and has great significance in the large-scale mining machinery industry.

## Figures and Tables

**Table 1:** The specification of the knock sensor used in actual machines.

Classification	Knock Sensor
Manufacturer	Continental Automotive
Model No.	Customized for use
Purpose	Shock events
Type	Piezoelectric (Flat response)
Frequency Range	3 to 26 kHz
Sensitivity	1.7 to 3.7 mV(m/s2) at 5 kHz Output@5 kHz + 15% at 8 kHz Output@5 kHz + 30% at 13 kHz Output@5 kHz + 100% at 18 kHz

**Table 2:** Parameter setting in each learning model.

Method	Specific Parameter
DCTLN [21]	$\lambda =\frac{2}{(1+\exp\left(-10\times p\right)}-1$
WDCNN [45]	λ=1, μ=100, σ=0.001
DANN [28]	$\lambda ,\;\mu =\frac{2}{(1+\exp{\left(-10\times p\right)}^{0.75}}$
DADA [46]	$\lambda =\frac{2}{(1+\exp\left(-10\times p\right)}-1$
Proposed model	Filters=32, 16, 8, kernel size=6, 3

**Table 3:** Parameters of the encoder in proposed model.

No.	Layer	Output Shape	Param #
1	Input	(None, 50, 513, 2)	0
2	Conv1d_0_1	(None, 50, 513, 32)	160
3	Maxpooling	(None, 50, 257, 32)	0
4	Dense_0	(None, 50, 257, 32)	1056
5	Multiply_0	(None, 50, 257, 32)	0
6	Conv1d_1_1	(None, 50, 257, 16)	1040
7	Maxpooling	(None, 50, 129, 16)	0
8	Dense	(None, 50, 129, 16)	272
9	Multiply_0	(None, 50, 129, 16)	0
	…	…	…
16	Conv1d_8_1	(None, 50, 130, 16)	272
17	Upsampling	(None, 50, 260, 16)	0
18	Conv1d_10_1	(None, 50, 258, 32)	1568
19	Upsampling	(None, 50, 516, 32)	0
20	Conv1d_12_1	(None, 50, 513, 2)	258

**Table 4:** Description of the CWRU dataset.

Dataset	A	B	C	D
Rotating Speed (rpm)	1797	1772	1750	1730
Load	0	1	2	3
Count of train data	12,540 (Respectively)			
Count of test data	3140 (Respectively)			

**Table 5:** The task of the CWRU dataset.

Class Label	0	1	2	3	4	5	6	7	8	9
Fault Location	NA	IF	BF	OF	IF	BF	OF	IF	BF	OF
Fault Size (mils)	0	7	7	7	14	14	14	21	21	21

**Table 6:** The comparison accuracy of the classification results with three new working condition domains on the CWRU dataset.

Task (Source→Target)	WDCNN		DANN		DADA		DCTLN		Without Attention		Proposed Model	
	Mean	Std	Mean	Std	Mean	Std	Mean	Std	Mean	Std	Mean	Std
A→B	71.47	0.01	67.76	0.01	63.27	0.01	56.23	0.06	62.14	0.01	84.43	0.01
A→C	72.87	0.02	68.96	0.01	62.86	0.01	57.78	0.01	68.07	0.01	86.43	0.01
A→D	71.90	0.01	69.81	0.00	66.97	0.00	54.11	0.02	67.54	0.01	85.67	0.01
B→A	69.91	0.01	66.73	0.01	67.82	0.01	52.56	0.01	68.14	0.00	84.67	0.00
B→C	67.48	0.01	64.96	0.01	70.95	0.00	56.67	0.01	62.14	0.01	85.35	0.01
B→D	67.58	0.01	69.65	0.01	68.93	0.01	58.12	0.01	67.48	0.00	82.43	0.00
C→A	68.88	0.01	59.70	0.00	62.99	0.01	59.12	0.02	69.47	0.01	83.34	0.01
C→B	70.15	0.00	64.40	0.01	59.55	0.00	54.32	0.01	68.11	0.01	82.45	0.00
C→D	65.83	0.01	69.82	0.00	59.62	0.01	52.13	0.01	67.45	0.00	82.21	0.01
D→A	68.14	0.01	58.62	0.01	57.89	0.01	57.45	0.03	56.47	0.01	81.24	0.01
D→B	65.17	0.01	57.41	0.01	62.53	0.01	58.23	0.04	53.88	0.01	80.67	0.01
D→C	69.90	0.01	58.98	0.01	59.90	0.01	46.12	0.01	52.54	0.01	80.67	0.01
AVG	69.11	0.01	66.15	0.01	63.61	0.01	55.24	0.02	63.62	0.01	83.63	0.01

**Table 7:** Description of the actual machine.

Dataset	A	B	C	D
Speed	100%	75%	50%	25%
Fault diameter (mm)	0.5, 1.0, 2.5	0.5, 1.0	0.5, 1.0, 2.5	0.5, 1.0
Train	14,688	3916	12,729	8486
Test	3671	979	3182	2121

**Table 8:** The comparison accuracy of classification results on the real machine dataset.

Task (Source→Target)	WDCNN		DANN		DADA		DCTLN		Without Attention		Proposed Model	
	Mean	Std	Mean	Std	Mean	Std	Mean	Std	Mean	Std	Mean	Std
A→B	67.47	0.02	68.76	0.03	67.27	0.08	52.24	0.02	62.14	0.07	83.76	0.08
A→C	72.87	0.03	72.96	0.08	70.86	0.03	50.4	0.01	65.80	0.09	85.16	0.05
A→D	71.90	0.08	73.81	0.12	68.97	0.02	55.28	0.23	67.54	0.12	83.23	0.02
C→A	62.88	0.08	71.70	0.07	72.99	0.12	50.32	0.11	69.47	0.01	78.21	0.03
C→B	73.15	0.03	74.40	0.10	72.55	0.09	50.52	0.13	67.11	0.03	79.56	0.07
C→D	68.83	0.02	68.82	0.11	69.62	0.07	51.36	0.23	65.45	0.07	82.56	0.09
AVG	69.52	0.04	71.74	0.09	70.38	0.07	51.68	0.12	66.25	0.07	82.08	0.06

## Data Availability

Data sharing not applicable.

## References

[1] Liao Y., Deschamps F., de Freitas Rocha Loures E., Ramos L.F.P. (2017). Past, present and future of Industry 4.0—A systematic literature review and research agenda proposal. Int. J. Prod. Res..

[2] Gao Z., Cecati C., Ding S.X. (2015). A survey of fault diagnosis and fault-tolerant techniques-Part II: Fault diagnosis with knowledge-based and hybrid/active approaches. IEEE Trans. Ind. Electron..

[3] Kim J.-Y., Cho S.-B. (2020). Deep CNN Transferred from VAE and GAN for classifying irritating noise in automobile. Neurocomputing.

[4] LeCun Y., Boser B., Denker J.S., Henderson D., Howard R.E., Hubbard W., Jackel L.D. (1989). Backpropagation Applied to Handwritten Zip Code Recognition. Neural Comput..

[5] Hochreiter S., Schmidhuber J. (1997). Long Short-Term Memory. Neural Comput..

[6] Miao H., Li B., Sun C., Liu J. (2019). Joint learning of degradation assessment and RUL prediction for aero-engines via dual-task deep LSTM networks. IEEE Trans. Ind. Informat..

[7] Wen S., Wang Y., Tang Y., Xu Y., Li P., Zhao T. (2019). Real-time identification of power fluctuations based on LSTM recurrent neural network: A case study on Singapore power system. IEEE Trans. Ind. Informat..

[8] Mbo’o C.P., Hameyer K. (2016). Fault Diagnosis of Bearing Damage by Means of the Linear Discriminant Analysis of Stator Current Features from the Frequency Selection. IEEE Trans. Ind. Appl..

[9] Wang H., Ren B., Song L., Cui L. (2020). A Novel Weighted Sparse Representation Classification Strategy Based on Dictionary Learning for Rotating Machinery. IEEE Trans. Instrum. Meas..

[10] Zhang B., Li W., Li X., Ng S. (2018). Intelligent fault diagnosis under varying working conditions based on domain adaptive convolutional neural networks. IEEE Access..

[11] Wang X., He H., Li L. (2019). A hierarchical deep domain adaptation approach for fault diagnosis of power plant thermal system. IEEE Trans. Ind. Inform..

[12] Zhang W., Li C., Peng G., Chen Y., Zhang Z. (2018). A deep convolutional neural network with new training methods for bearing fault diagnosis under noisy environment and different working load. Mech. Syst. Signal Process..

[13] Li X., Zhang W., Ding Q. (2019). Cross-domain fault diagnosis of rolling element bearings using deep generative neural networks. IEEE Trans. Ind. Electron..

[14] Li X., Zhang W., Xu N., Ding Q. (2019). Deep learning-based machinery fault diagnostics with domain adaptation across sensors at different places. IEEE Trans. Ind. Electron..

[15] Bu S.-J., Cho S.-B. (2020). Time series forecasting with multi-geaded attention-based deep learning for residential energy consumption. Energies.

[16] Yosinski J., Clune J., Bengio Y., Lipson H. How transferable are features in deep neural networks?. Proceedings of the 27th International Conference on Neural Information Processing Systems.

[17] Li X., Zhang W., Ding Q., Li X. (2019). Diagnosing rotating machines with weakly supervised data using deep transfer learning. IEEE Trans. Ind. Inf..

[18] He Z., Shao H., Jing L., Cheng J., Yu Y. (2020). Transfer fault diagnosis of bearing installed in different machines using enhanced deep auto-encoder. Meas. J. Int. Meas. Confed..

[19] Mao W., Ding L., Tian S., Ling X. (2019). Online detection for bearing incipient fault based on deep transfer learning. Measurement.

[20] Lu W., Liang B., Cheng Y., Meng D., Yang J., Zhang T. (2017). Deep model-based domain adaptation for fault diagnosis. IEEE Trans. Ind. Electron..

[21] Guo L., Lei Y., Xing S., Yan T., Li N. (2019). Deep convolutional transfer learning network: A new method for intelligent fault diagnosis of machines with unlabeled data. IEEE Trans. Ind. Electron..

[22] Gretton A., Borgwardt K., Rasch M., Schölkopf B., Smola A. (2012). A kernel two-sample test. J. Mach. Learn. Res..

[23] Patel V.M., Gopalan R., Li R., Chellappa R. (2015). Visual domain adaptation: A survey of recent advances. IEEE Signal Proces. Mag..

[24] Li X., Zhang W., Ding Q., Sun J.-Q. (2019). Multi-Layer domain adaptation method for rolling bearing fault diagnosis. Signal Process..

[25] Li X., Jiang H., Wang R., Niu M. (2021). Rolling bearing fault diagnosis using optimal ensemble deep transfer network. Knowledge-Based Syst..

[26] Wen L., Gao L., Li X. (2017). A new deep transfer learning based on sparse auto-encoder for fault diagnosis. IEEE Trans. Syst. Man Cybern. Syst..

[27] Li X., Jia X.-D., Zhang W., Ma H., Luo Z., Li X. (2020). Intelligent cross-machine fault diagnosis approach with deep auto-encoder and domain adaptation. Neurocomputing.

[28] Ganin Y., Ustinova E., Ajakan H., Germain P., Larochelle H., Laviolette F., Marchand M., Lempitsky V. (2016). Domain adversarial training of neural networks. J. Mach. Learn. Res..

[29] Tzeng E., Hoffman J., Saenko K., Darrell T. Adversarial discriminative domain adaptation. Proceedings of the IEEE Conference on Computer Vision and Pattern Recognition.

[30] Long M., Cao Z., Wang J., Jordan M.I. (2005). Conditional adversarial domain adaptation. Advances in Neural Information Processing Systems.

[31] Li X., Zhang W., Ma H., Luo Z., Li X. (2020). Deep learning-based adversarial multi-classifier optimization for cross-domain machinery fault diagnostics. J. Manuf. Syst..

[32] Liu Z.H., Lu B.L., Wei H.L., Wei H.L., Chen L., Li X.H., Ratsch M. Deep adversarial domain adaptation model for bearing fault diagnosis. IEEE Trans. Syst. Man Cybern. Syst..

[33] Han T., Liu C., Yang W.G., Jiang D.X. (2019). A novel adversarial learning framework in deep convolutional neural network for intelligent diagnosis of mechanical faults. Knowl.-Based Syst..

[34] Sun S.N., Yeh C.F., Hwang M.Y., Ostendorf M., Xie L. Domain adversarial training for accented speech recognition. Proceedings of International Conference on Acoustics, Speech, and Signal Processing.

[35] Weinberger K., Saul L. (2009). Distance metric learning for large margin nearest neighbor classification. J. Mach. Learn. Res..

[36] Köstinger M., Hirzer M., Wohlhart P., Roth P.M., Bischof H. Large scale metric learning from equivalence constraints, In Proceedings of the IEEE Conference on Computer Vision and Pattern Recognition, Providence, RI, USA, 16–21 June 2012, 2288–2295.

[37] Tran D., Sorokin A. Human activity recognition with metric learning. Proceedings of the European Conference on Computer Vision.

[38] Bahdanau D., Cho K., Bengio Y. Neural machine translation by jointly learning to align and translate. Proceedings of the International Conference on Learning Representations.

[39] Qin Y., Song D., Cheng H., Cheng W., Jiang G., Cottrell G. (2017). A dual-stage attention-based recurrent neural network for time series prediction. arXiv.

[40] Dadon I., Koren N., Klein R., Lipsett M.G., Bortman J. (2020). Impact of gear tooth surface quality on detection of local faults. Eng. Fail. Anal..

[41] LeCun Y., Bengio Y., Hinton G. (2015). Deep learning. Nature.

[42] Vincent P., Larochelle H., Bengio Y., Manzagol P.A. Extracting and composing robust features with denoising autoencoders. Proceedings of the 25th International Conference on Machine Learning.

[43] Berglund M., Raiko T., Honkala M., Kärkkäinen L., Vetek A., Karhunen J. (2015). Bidirectional recurrent neural networks as generative models. Proceedings of the Advances in Neural Information Processing Systems.

[44] Woo S., Park J., Lee Y.J., Kweon S.I. CBAM: Convolutional block attention module. Proceedings of the European Conference on Computer Vision.

[45] Zhang W., Peng G., Li C., Chen Y., Zhang Z. (2017). A new deep learning model for fault diagnosis with good anti-noise and domain adaptation ability on raw vibration signals. Sensors.

[46] Hui T., Kui J. (2019). Discriminative adversarial domain adaptation. arXiv.

[47] Smith W.A., Randall R.B. (2015). Rolling element bearing diagnostics using the Case Western Reserve University data: A benchmark study. Mech. Syst. Signal Process..

